# Advances in testing for sample manipulation in clinical and forensic toxicology—part B: hair samples

**DOI:** 10.1007/s00216-023-04706-7

**Published:** 2023-04-28

**Authors:** Dirk K. Wissenbach, Tina M. Binz, Andrea E. Steuer

**Affiliations:** 1 Institute of Forensic Medicine, Jena University Hospital, Jena, Germany; 2 Center for Forensic Hairanalytics, Zurich Institute of Forensic Medicine, University of Zurich, Zurich, Switzerland; 3 Department of Forensic Pharmacology & Toxicology, Zurich Institute of Forensic Medicine (ZIFM), University of Zurich, Winterthurerstrasse 190/52, CH-8057 Zurich, Switzerland

**Keywords:** Hair, Clinical toxicology, Forensic toxicology, Sample manipulation testing

## Abstract

As a continuation of part A, focusing on advances in testing for sample manipulation of urine samples in clinical and forensic toxicology, part B of the review article relates to hair, another commonly used matrix for abstinence control testing. Similar to urine manipulation, relevant strategies to manipulate a hair test are lowering drug concentrations in hair to undercut the limits of detection/cut-offs, for instance, by forced washout effects or adulteration. However, distinguishing between usual, common cosmetic hair treatment and deliberate manipulation to circumvent a positive drug test is often impossible. Nevertheless, the identification of cosmetic hair treatment is very relevant in the context of hair testing and interpretation of hair analysis results. Newly evaluated techniques or elucidation of specific biomarkers to unravel adulteration or cosmetic treatment often focused on specific structures of the hair matrix with promising strategies recently proposed for daily routine work. Identification of other approaches, e.g., forced hair-washing procedures, still remains a challenge in clinical and forensic toxicology.

## Introduction

Continuing on part A of the critical review article on advances in testing for sample manipulation in clinical and forensic toxicology, part B will focus on hair samples used in abstinence control testing. Hair has gained massive importance for retrospective consumption monitoring in recent years, mainly because of its noninvasive sampling, non-critical storage at room temperature, and long-term detection window. As substances are constantly incorporated into the growing hair shaft, with mean hair growth for head hair of 1 cm/month, segmented hair analysis often allows the detection of cumulative analyte levels corresponding to a particular hair growth period [1].

Manipulation strategies of hair samples mainly include lowering of drug hair concentration, either through forced washing-out attempts or by chemical treatment of the hair samples. For the latter, it remains impossible from an analytical point of view to differentiate between usual cosmetic hair treatment and deliberate manipulation to circumvent a positive drug test. However, in abstinence control programs, participants are usually instructed in advance not to treat their hair cosmetically, e.g., through bleaching. Further, adequate testing for such manipulation, whether they relate to deliberate drug removal from the hair matrix or usual, personal-inspired cosmetic treatment, is important for the interpretation of hair analysis results. While general issues and challenges regarding the interpretation of hair testing results—including cosmetic hair treatments—are extensively described in the literature [1–3], to the best of our knowledge, recent progress on testing for (deliberate) hair manipulation has not yet been reviewed. Following this, part B of the current review on sample manipulation will summarize the different strategies that might be used deliberately to avoid a positive hair drug test, and focus on and critically discuss recent innovations on their detection. A PubMed search was conducted for new and innovative technical developments or biomarkers within the past 10 years with the following search terms: “hair AND adulteration,” “hair AND tampering,” “bleaching AND hair AND drug detection,” “dye AND hair AND drug detection,” and “cosmetic treatment AND hair analysis.”

## Ways of hair manipulation

Different treatments might be intentionally used to circumvent positive drug tests. A brief overview of the studied manipulation strategies/cosmetic treatments is provided in Table 1 and will be briefly summarized in the following subchapters. In contrast to urine samples, where currently, next to sample dilution, particularly substitution with synthetic products plays a crucial role, hair substitution is not a highly relevant manipulation approach given the noninvasive, direct sampling process. Intentional hair manipulation to avoid positive drug testing mainly relies on forced washing-out effects (“dilution of the hair”), for example, through extensive washing or even the use of commercially available cleansing shampoos advertised to produce negative hair testing results. Cosmetic hair treatments are manifold and include chemical (alkaline relaxation) or thermal hair straightening, perming (permanent waves), or different types of coloration from natural products (henna), tinting (temporary coloration), to (semi)permanent dyes or bleaching. It is well known that already seasonal effects, e.g., in terms of ultraviolet (UV) light exposure (sunlight), may damage the hair shaft, which can reduce drug hair concentrations as well [4–6]. Overall, the extent to which drug concentrations change—most often decrease—as a consequence of (un)intentional cosmetic treatment depends not only on the drug stability itself but also on the initial drug concentrations and the properties of the hair matrix [1]. Morphological changes of the hair structure leading to porous or damaged hair can influence drug incorporation, fixation, or release from the keratin structures, for instance, increased leaching-out effect [7, 8].

**Table 1:** Impact on hair manipulation/cosmetic treatments on certain drugs

Manipulation/cosmetic treatment	Drugs investigated	Hair concentration change	Reference
Forced washing (chlorinated water, hair tonic, disinfectant, etc.)	EtG	Decrease (up to − 65%)	[9]
	DOA	Decrease (up to − 80%)	[10]
Cleansing shampoos (Ultra Clean, Ultra Klean, Folli-Kleen, Detox, High Voltage Detox Folli-cleans)	EtG	No significant effect vs. decrease (up to − 95%)	[11, 12]
	DOA	Decrease (up to − 72%)	[13, 10]
Henna coloration	EtG	Decrease (up to − 100%)	[14]
Tinting	EtG	Decrease (up to − 70%)	[14, 15]
	DOA	No significant effect	[16]
(Semi)permanent dying	EtG	Decrease (up to − 80%)	[14, 17, 18]
	DOA	Decrease (up to − 64%)	[7, 19, 19–21]
Bleaching	EtG	Decrease (up to − 82%)	[14, 18]
	DOA	Decrease (up to − 98%)	[16, 20, 22–24]
Perming	EtG	Decrease (up to − 100%)	[15]
	DOA	Decrease (up to − 100%)	[16, 20, 21, 23]
Straightening (thermal—200 °C, 30 × , 2 s; chemical)	EtG	Decrease (up to − 79%) vs Increase (up to 50.9%)	[25]
	DOA	Decrease (up to − 100%) Increase (up to > 600%, AEME, CBN)	[26, 26–28]

*EtG* ethylglucuronide, *DOA* drug of abuse, *AEME* anhydroecgonine methyl ester

### Washout effects (“dilution”)

Washout effects can already occur from daily hygiene. For instance, Luginbuehl et al. systematically studied the influence of normal water in contrast to chlorinated water on hair ethyl glucuronide (EtG) concentrations over up to 10 h incubation. They found EtG concentrations were reduced by 47% (32–60%) or 57% (52–65%), respectively [9]. Baeck et al. showed that ten times repeated washing indicated similar damage to the hair, with consequently similar decreasing concentrations of amphetamine and methamphetamine, as observed for a single hair dye [19]. For targeted drug removal, more and more “special detox shampoos” are being sold these days that promise to cleanse the hair before a hair test. While few studies found decreases in hair concentrations for several drugs [10, 13], Binz et al. found that EtG levels did not significantly change after a single application of different detox shampoos, according to the manufacturer’s instructions [11]. However, as recently published by Luginbuehl et al*.*, drug users seem to recommend not only a single use but a repetitive, prolonged application [12]. Experiments, with up to 10 h of incubation, revealed significant EtG loss in only one (high-voltage detox Folli-cleanse) of four tested products, with mean decreases of 73% [12]. Regarding tetrahydrocannabinol (THC), other everyday products such as vodka, disinfectants, or hair tonics reduced THC hair concentrations in similar ranges as a special detox shampoo [10].

### Cosmetic treatment ((un)intentional adulteration): coloration (bleaching, dying)

Temporary hair coloration products such as tinting achieve a coloring effect directly through coloring ingredients that only dye the hair cuticula and, as such, are easily washed out after a few hair washes. In contrast, most (semi)permanent products contain alkali solutions and colorless precursors of low molecular weight in combination with oxidizing agents. Alkali solutions most often consist of ammonia, which accelerates hair swelling and facilitates diffusion of the present color precursors in and outside the hair cortex. Depending on the extent of cortex penetration—typically shallow for semi-permanent and deep for permanent dyes—the color effect lasts for a few weeks or indefinitely, respectively. The color itself then appears following an oxidative reaction of these precursors in the hair cortex, which is typically achieved by the addition of H_2_O_2_ in concentrations of 2% (semi-permanent dye) to 6% (permanent dye), respectively [14, 29]. The bleaching (decoloration) formula contains 6–12% hydrogen peroxide combined with a booster such as ammonium or potassium persulfate [30]. Particularly bleaching, with the highest H_2_O_2_ concentrations, has been shown to cause oxidative damage and degradation of melanin granules [7]. Next to these chemical hair coloring methods, natural products exist. The main ingredient of henna, probably the most common natural hair dye, is 2-hydroxy-1,4-napthoquinone (lawsone), which colors skin or hair red without using hydrogen peroxide or other oxidants. The effects of different coloring procedures have been tested extensively in vitro for EtG and various drugs (Table 1). Even coloration with henna, which did not contain any oxidants, and temporary dying, such as tinting, revealed significant decreases in EtG concentrations in one study [14]. At the same time, no effects were observed in other experiments on EtG [15] or for methamphetamine [16] when hair samples were temporarily dyed. Following (semi)permanent coloration, all drugs decreased in concentration, except for cannabinoids, which showed only slightly reduced hair concentrations following permanent dyeing [20]. Even complete degradation resulted from bleaching. Overall, in vitro studies revealed that the H_2_O_2_ concentration represents a decisive factor in the degree of hair damage and degradation/decrease of hair drug concentrations.

In contrast to the majority of studies done by in vitro experiments, Agius et al. statistically compared the positivity rate for EtG and other drugs in untreated or in vivo treated (dyed or bleached) hair samples in a large population. Interestingly, the positivity rate for none of the drugs or EtG routinely tested in cosmetically treated authentic samples (*n* = 1026) was significantly lower than the positivity rate for the same drugs in nontreated hair samples (*n* = 9488) [31]. However, other studies focusing on authentic hair treatments (in vivo treatments) [7, 17, 18] were mainly in line with results from the aforementioned in vitro experiments, showing marked decreases in EtG and drug concentrations. In authentic samples, differentiation between permanent dying and bleaching is difficult because previous bleaching cannot be excluded entirely, even in cases of conspicuous hair coloration [31].

### Cosmetic treatment ((un)intentional adulteration): perming

Perming involves a two-step procedure consisting of a combination of alkaline thioglycolate, which opens the hairs’ cuticle and reduces disulfide bonds, followed by their reforming in curls with low concentrations of H_2_O_2_ (2%) [16, 30]. Hair concentrations of EtG, as well as those of the investigated drugs amphetamine/methamphetamine, morphine/codeine/dihydrocodeine, and cannabinoids including THC, THC-COOH, cannabinol (CBN), and cannabidiol (CBD), clearly decreased following permanent waving procedures (Table 1).

### Cosmetic treatment ((un)intentional adulteration): straightening

Hair straightening, often through extreme heat application (thermal straightening), has become a regular, daily cosmetic treatment. Iron plates are usually heated to temperatures between 150 and 250 °C. The breakup of hydrogen and disulfide bonds in the hair matrix allows keratin chains to move and assume a position resulting in temporarily straightened hair [26]. A relatively high variation, including decreases and increases in drug concentrations, has been observed following thermal straightening. These effects were not only related to the drug itself but seem to depend on hair color and/or general hair structure [25]. Particularly, benzoylecgonine and CBN concentrations clearly increased, most likely caused by the thermal conversion of cocaine and THC, respectively [26]. Also, anhydroecgonine methyl ester (AEME), a common marker for smoked cocaine (“Crack”) consumption, was shown to be produced under conditions used for thermal straightening [27]. Chemical straightening products, also referred to as alkaline relaxing, contain an alkaline agent, sodium, lithium, or potassium hydroxide (lye relaxer) or guanidine hydroxide (no-lye relaxer). Independent of the alkaline reagent used, drug concentrations were significantly reduced by this treatment [28].

## Current strategies for the detection of cosmetic treatment

To the best of our knowledge, no standardized, routine workflow to objectively screen for cosmetic treatment(s) or extensive hair washing exists (yet). Cleansing shampoos or extensive washing cannot be detected with the available methods. The current approach to identifying coloration or bleaching relies on case information and visual inspection of colored hair samples or extracts following standard sample preparation in the laboratory before analysis. The latter usually works for colored hair specimens, while after bleaching, extracts are almost colorless [32]. However, in the last 5 years, research efforts focused on new techniques and biomarker searches as tools for more objective markers of cosmetic hair treatment/hair adulteration, as detailed in the next chapter.

## New strategies to detect cosmetic hair treatment (adulteration)

Considerable research efforts were made to evaluate different cosmetic hair products, particularly their effect on the hair structure related to the damage they cause to the hair matrix, detection of the extent of hair damage, and improvement of respective cosmetics [33, 34]. Here, we will focus on advances in detecting cosmetic hair treatment/hair adulteration in the context of drug testing in clinical and forensic toxicology. An overview of the chosen techniques, biomarkers, or strategies, including details on the analytical parameters, is provided in Table 2.

**Table 2:** Method details of new approaches to screen for hair sample adulteration attempts/detection of cosmetic treatment

Matrix	Biomarker/technique	Sample preparation	Analytical device	Settings	Data evaluation	
Hair	Infrared spectroscopy (cysteic acid)	Pulverization	ATS-IR Bruker ATR Diamond Golden Gate accessory / IFS66v FTIR Vacuum spectrometer	Scan range 4000 and 600 cm^−1^ resolution 2 cm^−1^	Origin 9.0	[35]
	Infrared spectroscopy Additional analysis of cosmetic hair residues on the hair surface	None	ATS-IR Nicolet 6700 FTIR Spectrometer / continuum FTIR microscope Homemade slide-on GeμIRE accessory	256-scan condition Scan range 4000–650 cm^−1^ Resolution of 4 cm^−1^		[36]
	Fluorescence spectroscopy (oxidation of melanin)	3-cm segments Glycerol embedding on microscopic slide	Fluorescence microscope Axiostar plus, Zeiss	Mercury lamp 3 different excitation-emission filters - 365/12 nm–397 nm - 450–490 nm–515 nm - 546/12 nm–590 nm	Olympus SC20 camera (photographs)	[37]
Cosmetic products	Thioglycolic acid	Dilution	CE-PDA P/ACEMDQ system Beckman Instruments	Uncoated fused-silica capillary constant voltage of − 5 kV	32Karat software, 8.0 Quantification	[38]
Hair	Lawsone (henna)	Washing snippets (< 5 mm) Incubation / extraction H_2_O 24 h, 37 °C, water bath 1 h, ultrasonication	LC-DAD Agilent G1315A	Kinetex C18 column - 20 mM phosphate (pH 2.1) - ACN UV spectra 200–400 nm 280 nm for quantitative analysis	ChemStation Quantification 6-point calibration	[39]
Solution	Methamphetamine oxidation products		HPLC–MS/MS M-8000 (Hitachi) ion trap	ODS column - 10 mM NH4Ac - MeOH (17:3, v/v) ESI + / ESI −	LC/3DQMS system manager Mass spectra interpretation	[40]
Hair	Doxylamine oxidation product (doxylamine-N-oxide)	Washing Pulverization Incubation MeOH 2 h, 45 °C Sonication	UPLC-MS/MS Xevo TQS tandem MS ESI + , MRM	Acquity UPLC BEH C18 - H_2_O, 0.1% FA - ACN	Quantification 8-point calibration	[41]
Hair	Hair digestion	None		Non-proteolytic digestion system - 0.3% dithiothreitol, KOH pH 9.5 - 37 °C water bath, 2 h	Dissolved samples indicative for damaged hair	[8]
				proteolytic digestion system - 0.6% dithiothreitol, pH 9.5 - 0.25 unit/mL Proteinase K - 37 °C, 2 h	Visual inspection at 15-min intervals - intact hair: 2 h to dissolve - damaged hair faster - assignment of integrity values	
				Measurement of protein leakage in non-proteolytic digestion -0.3% dithiothreitol, KOH pH 9.5 - 37 °C water bath, 2 h - Bradford protein assay	Protein quantification	
Hair	PTCA	Washing Incubation/extraction (HCl) 24 h, 37 °C water bath 2 h ultrasonication Addition of ethyl acetate	LC-MS/MS Agilent G6460	Polaris C8‐A - 5 mM NH4COOH, 0.01% FA - ACN, 0.1% FA ESI − (PTCA), ESI + (diazepam-d5, IS) MRM	Agilent Mass Hunter Software (version B.07.00) Quantification 7-point calibration (PTCA-free albino rabbit hair)	[32]
		Washing snippets (2–4 mm) Incubation (HCl) 18 h, 45 °C Addition of ethyl acetate	UHPLC-MS/MS Sciex 5500 QTrap	Luna Omega Polar C18 column - 0.1% FA - ACN, 0.1% FA ESI − (PTCA), ESI + (diazepam-d5, IS) MRM	MultiQuant 2.1.1 Quantification 8-point calibration (PTCA-free albino rabbit hair)	[42]
		Washing Snippets (2–4 mm) Incubation/extraction (H_2_O/meoh 88:2, v/v) Overnight, room temp 2 h, ultrasonication	HPLC–MS/MS Sciex 4000 QTRAP	Luna Omega Polar C18 column - 0.1% FA - ACN, 0.1% FA ESI − MRM	MultiQuant 2.1.1 Quantification 6-point calibration (PTCA-free albino rabbit hair)	[43]
	PTeCA	Washing Pulverization Incubation/extraction (ACN/H_2_O (2:8, v/v)) 16 h, ultrasonication	LC-HRMS Sciex 6600 QTOF	XSelect HSST RP-C18 column - 10 mM NH4COOH, 0.1% FA -MeOH, 0.1% FA ESI- fullscan, DDA (separate)	MultiQuant 2.1 Semi-quantitative (peak area comparison)	[44]
Hair	Amino acids, lipids		UPLC-PDA UPLC-MS/MS		Metabolomics tools PCA, OPLS-DA	[45]
	Amino acids and hair damage markers: Alanine, arginine, aspartic acid, cysteic acid, cysteine, glutamic acid, glycine, histidine, 3-hydroxy kynurenine, isoleucine, kynurenine, lanthionine, leucine, lysine, methionine, phenylalanine, proline, serine; threonine, tyrosine, valine	Whole hair or 0.5-cm hair pieces HCl 24 h, 110 °C, Heated oven Dilution in H_2_O (10,000-fold)	LC-MS/MS Sciex 6500 + QTRAP	Phenomenex Luna Omega C18 Polar - H_2_O, 0.1% FA - ACN, 0.1% FA ESI + , MRM	Sciex OS 1.5 MQ Quantification 7-point calibration	[46]
	Untargeted search	Washing Pulverization Incubation/extraction (ACN/H_2_O (2:8, v/v)) 16 h, ultrasonication	LC-HRMS Sciex 6600 QTOF	XSelect HSST RP-C18 column - 10 mM NH4COOH, 0.1% FA - MeOH, 0.1% FA SeQuant ZIC HILIC - 25 mM NH4Ac, 0.1% AA - ACN, 0.1% AA ESI + , ESI − Full scan, DDA (separate)	Progenesis QI Metabo Analyst 4.0 Identification in Progenesis Qi (NIST, Metlin, HMDB) Identification in Peak View (in-house database)	[47]

*LIME* local interpretable model-agnostic explanations, *PDA* photodiodearray

Many newly evaluated techniques or biomarkers target specific structures of the hair matrix. Briefly, human hair is a protein filament built in three layers: cuticle, cortex, and medulla. It is primarily comprised of keratin but also contains lipids, water, trace elements, and pigments. Keratin is built from amino acids, with cysteine making up approximately 18% [48–50]. Melanin, which is only present in the cortex and medulla but not in the cuticula, is the pigment that causes the natural coloring of hair and consists of two types of polymeric macromolecules, eumelanin, and pheomelanin. Eumelanin gives the hair a black or brownish color and is built from tyrosine derivatives, mostly 5,6-dihydroxyindole (DHI) and 5,6-dihydroxyindole-2-carboxylic acid (DHICA), followed by polymerization [51]. Pheomelanin is a sulfur-containing compound with a yellow to reddish color shade [30, 52–54].

### Technical advances

In the technical field, there have been developments primarily in the use of infrared spectroscopy as well as fluorescence microscopy to detect oxidative hair treatments. Ammann et al. evaluated the impact of increased exposure to H_2_O_2_ in correlation to the EtG content in hair samples in a non-destructive way by attenuated total reflectance Fourier transform infrared spectroscopy (ATR-FTIR). Hair samples were bleached in vitro (10% H_2_O_2_), and the absorbance was measured in pulverized hair samples. Treated hair samples were shown to consistently display a significantly increased absorbance associated with the formation of cysteic acid, the oxidation product of the amino acid cysteine. Detection of cysteic acid proved to be rapid and sensitive enough to flag hair samples as bleached before the EtG levels were severely affected. As untreated hair contains minor amounts of cysteic acid and traces of other cysteine oxidation products, most likely caused by UV-light exposure or general weathering effects, semi-quantification and cut-off definition were required. The method has yet to be applied to other hair treatments and/or varying H_2_O_2_ concentrations [35]. Expanding on this, the ATR-FTIR technique was combined with a unique, homemade dome-shaped internal reflection element (Ge μIRE) accessary to detect cosmetic residues on single human hair samples [36]. This sampling technique—referred to by the authors as “Contact-and-Collect”—can transfer residual materials on the hair surface to the tip of the Ge μIRE. Oxidative treatment and perming by thioglycolate were shown to result in distinguishable IR spectra. However, validation or proof of applicability in (forensic) authentic cases was not performed (yet) [36].

Witt et al. employed fluorescence microscopy to distinguish natural hair from hair samples treated with different cosmetics. The hair pigment melanin is known to exhibit autofluorescence, but as a weak emitter, fluorescence detection is generally difficult. However, fluorescence considerably increases under oxidative conditions. Except for autofluorescence of melanin, no fluorescence was detectable in untreated hair, and no changes were observed by either tinting or natural cosmetics (henna) that lack oxidative agents. In contrast, coloration or bleaching with oxidative ingredients markedly increased fluorescence detection. The final applicability of this procedure was demonstrated in authentic, forensic cases with congruent analytical findings to respective self-reports on prior cosmetic treatment [37]. Next to melanin, tryptophan and its degradation products, kynurenine and formylkynurenine [55, 56], exhibit fluorescence. As such, hair samples with low levels of melanin, e.g., light-blond hair, can exhibit fluorescence which should allow the detection of potential adulteration by oxidative treatment. On the other hand, the fluorescence of tryptophan was shown to increase following treatment with reducing agents such as thioglycolate, which would also allow for the detection of perming [57]. In general, fluorescence microscopy poses a fast and economical technique to screen for oxidative hair treatment [37], but it may not be routinely available in forensic toxicological laboratories [32]. The same applies to the aforementioned infrared spectroscopy.

### Direct approaches

Hyphenated techniques with different chromatographic systems followed by diode array detection (DAD) or MS detection are much more common in toxicological laboratories. Only two direct approaches targeting unique ingredients of cosmetic products have been described with either CE-DAD or LC-DAD detection. Thioglycolic acid, as an ingredient/marker for permanent waving, was determined by a newly developed CE-DAD method cross-validated to former HPLC and ion chromatography methods and successfully applied to the analysis of 85 commercial depilatory creams and hair cosmetics. However, this method tested no authentic hair samples for thioglycolic acid [38]. Petzel-Witt et al. developed and validated an LC-DAD method for directly detecting lawsone, the principal coloring ingredient in henna. Initial attempts to use LC-MS caused by coagulation or polymerization of lawsone-containing solutions during ionization resulted in blockage of the skimmer. In 12 henna-colored hair samples, a median lawsone concentration of 92.6 ng/mg (range 27.3–253.7 ng/mg) was detectable. Furthermore, the method was successfully applied to actual cases and allowed the identification of a suspicious hair sample from a drug testing program as Henna-colored [39].

### Indirect approaches: strategies to identify damaged hair

Hill et al. tested three different methods to identify a hair sample as porous or damaged, for example, as a consequence of any treatment procedure. First, the digestion rates of hair in dithiothreitol with (proteolytic) and without proteinase K (non-proteolytic) were determined. In the first approach, the status of the hair samples was visually inspected after a 2-h incubation (non-proteolytic), and only exceptionally damaged hair samples were expected to be dissolved. Following the proteolytic method, hair samples were evaluated in 15-min intervals, and the time at which the sample dissolved performed as a marker for integrity. In addition, a protein measurement method applied to dithiothreitol-digested (non-proteolytic) samples was tested, which determines the amount of solubilized proteins by the Bradford protein assay, with higher protein levels expected from porous and damaged hair samples. The applicability of these methods was tested and shown for permed and bleached hair, while chemically relaxed hair did not dissolve even over 24 h. However, this characteristic of relaxed hair can be considered a test for its identification since normal hair dissolves within 120 min. Overall, the proteolytic test provided a more precise estimation of porosity [8].

### Indirect approaches: oxidation markers of drugs

Similar to advances in urine testing for chemical sample adulteration, attempts have been made to detect oxidized drugs (metabolites) or changes in endogenous hair components in hair analysis. Tanaka et al. investigated the oxidation of methamphetamine in the presence of H_2_O_2_ as the main ingredient of dye or decolorants. Ortho-, meta- and para-hydroxy methamphetamine were detected next to methamphetamine N-oxide and N-hydroxy methamphetamine. However, the study was never continued beyond the investigation of solutions, and detectability in authentic hair samples has yet to be discussed [40]. Doxylamine concentrations in authentic hair samples decreased through oxidative dyeing procedures, while an increase in the oxidation product N-doxylamine-oxide was observed [41]. As N-doxylamine-oxide is also an in vivo metabolite of doxylamine, its presence in hair samples cannot be directly related to hair adulteration.

### Indirect approaches: oxidation of endogenous compound markers

**Fig. 1:**
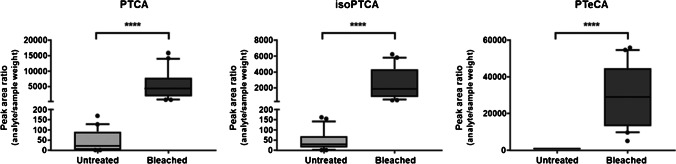
Box plots for PTCA, iso-PTCA, and PTeCA. Analyte peak area ratios (compound/sample weight) are depicted for untreated hair samples (light gray) and hair samples after treatment with 9% H_2_O_2_ for 30 min (dark gray), *n* = 21 for each group (experiment 1). Statistical analysis performed with a paired *t* test: ****P < 0.0001. Reproduced from Eisenbeiss et al. [44] with permission from WILEY

Recently, the oxidation of common hair constituents, such as melanin, gained attention as a potential strategy for detecting oxidative hair treatment. 1H‐Pyrrole‐2,3,5‐tricarboxylic acid (PTCA) is the primary degradation product of eumelanin, whereas aminohydroxphenylalanine isomers (AHP) are formed as degradation products of pheomelanin [58]. Several studies on melanin degradation found next to PTCA other pyrroles, such as 1H-pyrrole-2,3-dicarboxylic acid (PDCA), 1H-pyrrole-2,3,4-tricarboxylic acid (iso-PTCA), and 1H-pyrrole-2,-3,4,5-tetracarboxylic acid (PTeCA), that can be formed during alkaline H_2_O_2_ oxidation either by direct DHI- or DHICA oxidation or via cross-linking of DHI moieties [51, 54, 59, 60]. A first,application in the framework of drug testing was described by Petzel-Witt et al., who developed and validated an LC-MS/MS method to determine PTCA in hair samples. The influence of H_2_O_2_ in varying concentrations and incubation time on the formation of PTCA was tested. The effects of common hair dyes evaluated on in vitro—treated authentic hair samples. As expected, PTCA formation depended on both incubation time and concentration. PTCA could also be detected in natural hair samples, with a mean concentration of 8.4 ng/mg (< 2.1–16.4 ng/mg). A cut-off of 20 ng/mg was proposed for the differentiation between treated and natural hair. As expected, cosmetic products free of H_2_O_2_, like henna or temporary dyes, did not influence hair PTCA levels, while significant increases were measurable following (semi)permanent dyeing or bleaching. Given the dependency of PTCA increases on the melanin content of the hair, white or light-blond samples might not result in sufficient PTCA increases, and adulteration might remain unidentified by this method. Casati et al. continued the suitability evaluation of PTCA in larger cohorts by the determination of its baseline levels in hair samples from six ethnic subgroups, African, Arab, Asian-Pacific, Caucasian, Hispanic, and Indian (*n* = 156, hair color from dark brown to black) and after treatment with professional bleaching products (12% H_2_O_2_). Quantified baseline levels were in line with those published by Petzel-Witt [32], ranging from 0.44 to 23.7 ng/mg, with no observed statistically significant differences between ethnic subgroups, except for Caucasians that exhibited lower PTCA concentrations. In vitro bleached hair showed increased PTCA concentrations from 4.16 to 32.3 ng/mg [42]. Analysis of over 1000 authentic hair samples classified according to natural or in vivo–treated hairs by self-reports revealed PTCA concentrations of 0.009 to 49.8 ng/mg (mean 0.66 ng/mg, median 0.02 ng/mg). Only 60 subjects declared cosmetic hair treatment and were shown to have significantly higher measurable PTCA levels compared to the natural hair group. Gender-related differences were observed for PTCA baseline levels but not for concentrations observed after cosmetic treatment. PTCA could be considered a suitable marker to detect bleaching or oxidative hair treatment, regardless of gender [43]. The other described melanin degradation products—PDCA, iso-PTCA, and PTeCA—were initially claimed as unsuitable biomarkers for oxidative treatments, as they were considered to occur in low amounts [32]. Interestingly, their targeted evaluation within the framework of a larger untargeted data acquisition project (quadrupole time of flight, qTOF, analysis) of bleached hair samples also revealed significantly elevated concentrations of iso-PTCA and particularly PTeCA as shown in Fig. 1 already with low H_2_O_2_ concentrations and after short incubation times. In this regard, initial studies suggested that PTeCA was detectable only after oxidative treatment, as demonstrated in 12 of 13 authentic in vivo bleached hair samples, but remained undetectable in untreated hair segments of up to 12 cm from the root [44]. PTeCA is not yet available as a reference compound, though, and quantitative levels need to be evaluated in larger study cohorts and with more sensitive methods, e.g., using triple quadrupoles. If future work confirms exclusive PTeCA formation after oxidative treatment, PTeCA might be superior to PTCA for routine analysis as it omits a cut-off decision. However, in line with PTCA, the issue of melanin dependency persists and bears the risk that adulteration in naturally white or light-blond hair might go unnoticed.

Next to melanin, changes in other common hair structures like amino acids as the building blocks of keratin and several lipids were investigated to observe hair damage and/or as potential adulteration or cosmetic treatment markers [45, 46]. Joo et al. comprehensively investigated the alteration of amino acids, including cysteic acid and different lipids (ceramides, free fatty acids, cholesterol, and 18-methyl eicosanoic acid) after chemical treatment with LC-photodiode array and LC-MS/MS analysis. They used multivariate statistical tools to recognize global alterations and revealed that the combined assessment of cysteic acid, the ratio of cysteine/cysteic acid, tryptophan, and methionine are useful in grading hair damage caused by chemical treatment [45]. Similarly, Gargano et al. quantified 21 analytes, generally amino acids and four hair damage biomarkers in one LC-MS/MS method, including an isotope-dilution strategy. This method was applied to two commercial hair sample batches after six different in vitro treatments (UV irradiation, light, and strong bleach, heat treatment, and glyoxylic acid treatment before straightening). In line with Joo et al*.*, cysteine, cysteic acid, methionine [45], lanthionine, kynurenine, and lysine responded to one or more of the treatments and allowed discrimination of treated hair samples. Lanthionine was described as a biomarker for high-temperature treatments, supposed to occur from heat-induced beta-elimination of cysteine and following Michael addition of cysteine [46].

Instead of a targeted evaluation of highly abundant constituents of human hair, Eisenbeiss et al. used an untargeted metabolome strategy to screen beyond the expected melanin, amino, or fatty acid hair constituents or their degradation products for possible biomarkers of oxidative treatment (bleaching). Following metabolomics-like data evaluation, 69 metabolites were identified that significantly changed between natural and bleached hair samples. Mainly decreases in amino acids, carnitines, purines, nucleosides, nucleotides, and acetylneuraminic acid could be detected. Identifying already known formation/increases of melanin and cysteine degradation products proved the general applicability of the approach. Except for PTeCA, no new compounds that were exclusively formed through the oxidation process could be identified. However, identification issues were identical to those already described for urine metabolome analysis. Also, only a few compounds, including some carnitines, and in line with former urine analysis, uric acid and acetylneuraminic acid wholly degraded. Uric acid oxidation products found in treated urine samples remained undetectable in the hair. As most of the identified compounds decreased in concentration but remained detectable, different strategies were evaluated to distinguish treated from untreated samples without requiring quantification and tedious cut-off definition. Discrimination power could be substantially improved by forming metabolite ratios of elevated over lowered amino acids, e.g., for the pair tyrosine/cysteic acid [44]. Again, further applications to different hair treatments (in vivo) are still pending.

## Summary and critical evaluation

Despite the inability to clearly distinguish between deliberate hair manipulation to avoid positive drug test results, and usual (often daily) cosmetic hair treatment, hair manipulation/cosmetic hair treatment remains an important issue in clinical and forensic toxicology. Particularly so-called cleansing shampoos are certainly intended and advertised to be used to remove drugs from the hair. Up to now, no standardized, routine workflow to objectively screen for cosmetic treatment(s) or extensive hair washing exist (yet). While hair dying is typically visually inspected in the lab—either on the hair itself or through obvious coloration of the sample extracts—cleansing shampoos or extensive washing cannot be detected at all. Major progress has been made in applying different techniques, such as IR and fluorescence microscopy, or search for new, more objective biomarkers to detect cosmetically treated/chemically adulterated hair. Overall, especially the proposed biomarker PTCA has been thoroughly investigated and can be included into routine hair testing procedures to screen for oxidative hair treatments. As a small molecule, common available infrastructure in toxicological laboratories is feasible for its detection. Still, PTCA bears some limitations given its presence also in native hair samples and its dependency of the hair’s melanin content (hair color). In contrast to the comparatively simple implementation of PTCA detection, techniques such as IR or fluorescence microscopy might complement screening for hair manipulation, but may not be routinely available in clinical/forensic toxicological laboratories.
